# Identification of a nomogram based on an 8-lncRNA signature as a novel diagnostic biomarker for head and neck squamous cell carcinoma

**DOI:** 10.18632/aging.104014

**Published:** 2020-10-22

**Authors:** Rui Mao, Yuanyuan Chen, Lei Xiong, Yanjun Liu, Tongtong Zhang

**Affiliations:** 1 Affiliated Hospital of Southwest Jiaotong University, Chengdu 610036, China; 2 Department of Pathology, The Third People’s Hospital of Chengdu, Chengdu 610031, China; 3 Department of Otolaryngology, The Third People’s Hospital of Chengdu, Chengdu 610031, China; 4 The Center of Gastrointestinal and Minimally Invasive Surgery, The Third People’s Hospital of Chengdu, Chengdu 610031, China; 5 Medical Research Center, The Third People’s Hospital of Chengdu, The Affiliated Hospital of Southwest Jiaotong University, The Second Chengdu Hospital Affiliated to Chongqing Medical University, Chengdu 610031, Sichuan, China

**Keywords:** head and neck squamous carcinoma, long noncoding RNAs, prognosis, bioinformatics, qRT-PCR

## Abstract

Long noncoding RNAs (lncRNAs) have been proposed as diagnostic or prognostic biomarkers of head and neck squamous carcinoma (HNSCC). The current study aimed to develop a lncRNA-based prognostic nomogram for HNSCC. LncRNA expression profiles were downloaded from The Cancer Genome Atlas (TCGA) database. After the reannotation of lncRNAs, the differential analysis identified 253 significantly differentially expressed lncRNAs in training set TCGA-HNSC (n = 300). The prognostic value of each lncRNA was first estimated in univariate Cox analysis, and 41 lncRNAs with P < 0.05 were selected as seed lncRNAs for Cox LASSO regression, which identified 11 lncRNAs. Multivariate Cox analysis was used to establish an 8-lncRNA signature with prognostic value. Patients in the high-signature score group exhibited a significantly worse overall survival (OS) than those in the low-signature score group, and the area under the receiver operating characteristic (ROC) curve for 3-year survival was 0.74. Multivariable Cox regression analysis among the clinical characteristics and signature scores suggested that the signature is an independent prognostic factor. The internal validation cohort, external validation cohort, and 102 HNSCC specimens quantified by qRT-PCR successfully validate the robustness of our nomogram.

## INTRODUCTION

The incidence and mortality of head and neck cancer have increased dramatically in recent decades. Most of the patients present advanced diseases with the characteristics of early invasion and metastasis [1, 2] [3]. Besides, despite advances in treatment, the 5-year survival rate for head and neck cancer remains around 60%, which has improved only slightly over the past few decades [3, 4]. The current prognostic models for patients with HNSCC are based on clinicopathological parameters, but many cases with the same clinical stage show different results [2, 5]. Therefore, for patients with HNSCC, there is an urgent need for a useful prognostic model that can predict the survival and prognosis of patients.

To identify lncRNAs associated with prognosis in HNSCC, we integrated gene matrix and clinical information from a TCGA dataset and the GSE65858 dataset to establish a nomogram with 8-lncRNA signature. Functional enrichment and WGCNA were performed to predict the potential functions of the gene modules, which are both related to the lncRNAs and clinical characteristics.

## RESULTS

### Preprocessing of the data sets

We downloaded the gene matrix of 546 samples from the TCGA-HNSC database, which included 502 tumour and 44 normal samples. We divided all HNSCC patients with complete information (n=499) in TCGA-HNSC into training cohort and validation cohort, in a random manner according to a ratio of 3:2.

Moreover, From May 2017 to August 2018, a total of 102 frozen, surgically resected tumor tissues were obtained from patients with pathological diagnosis of HNSCC at Chengdu Third People's Hospital. The specimens were frozen with liquid nitrogen immediately after removal and transferred to the −80°C refrigerator.

### Differential analysis

**Figure 1:**
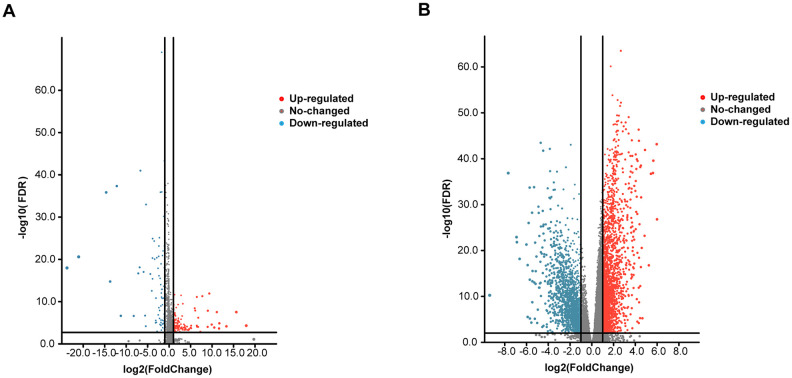
**Volcano plot of the differentially expressed mRNAs and lncRNAs between HNSCC and para-carcinoma tissues.** Red indicates high expression, and blue indicates low expression (|log2FC| > 1 and *P* value < 0.05). The Y axis represents adjusted *P* values, and the X axis represents log2FC values. The RNAs studied in this article have been marked in the figure. (**A**) Volcano plot of the differentially expressed lncRNAs. (**B**) Volcano plot of the differentially expressed mRNAs.

We conducted a differential analysis of the 300 tumor and 44 normal samples. Eventually, we obtained a total of 19754 mRNAs and 14847 lncRNAs. After obtaining the expression data, we identified differentially expressed genes using the software package EdgeR, selecting genes that had at least 2-fold higher expression levels in HNSCC samples (Poisson model FDR < 0.05). Therefore, after screening, we obtained 4150 reliably expressed mRNAs and 253 lncRNAs (Figure 1A, 1B).

### Identification of 8-lncRNAs for predicting HNSCC patient survival

**Figure 2:**
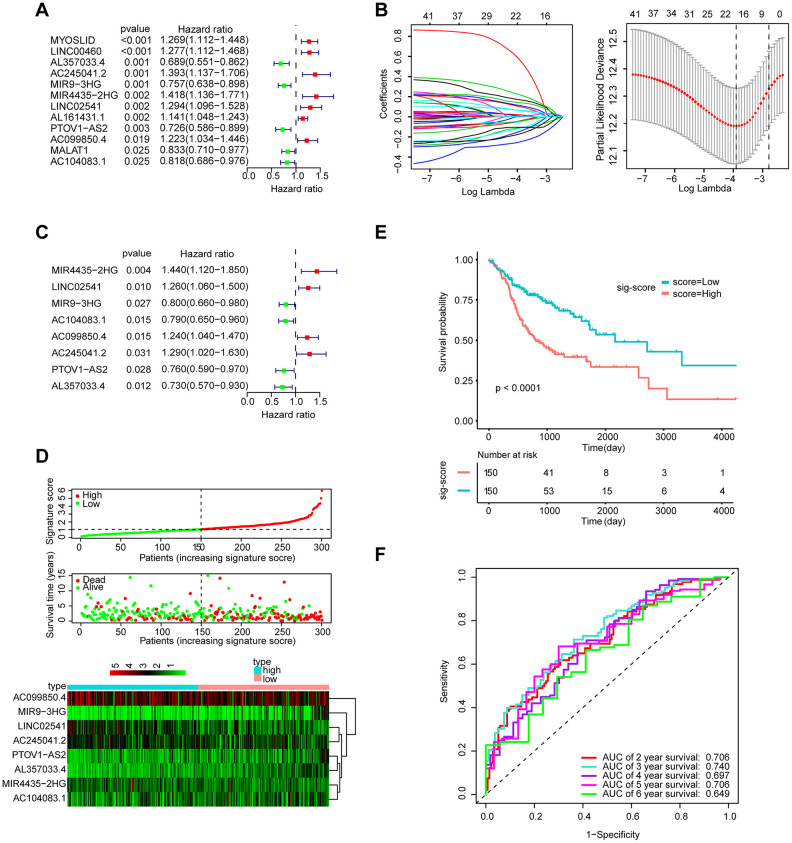
**Establishment and validation of the eight-lncRNA prognostic signature.** (**A**–**C**) The procedure of establishing the prognostic signature. (**D**) Correlation between the prognostic signature and the overall survival of patients in the TCGA cohort. The distribution of signature scores (top), survival time (middle) and lncRNA expression levels (bottom). The black dotted lines represent the median signature score cut-off dividing patients into the low- and high-signature groups. The red dots and lines represent the patients in the high-score group. The green dots and lines represent the patients in the low-score group. (**E**) Kaplan-Meier curves of OS based on the 8-lncRNA signature. (**F**) ROC curve analyses based on the 8-lncRNA signature.

A total of 253 lncRNAs with significant differences were identified to have prognostic significance in univariate Cox survival analysis, and 41 with P < 0.05 were screened out and applied in the following analysis (Figure 2A). As shown in Figure 2B, 2C, LASSO regression analysis identified 11 lncRNAs (lambda value=11), which were then used in the multivariate Cox regression. Finally, 8 lncRNAs for predicting HNSCC patient survival were identified, including MIR4435-2HG, LINC02541, MIR9-3HG, AC104083.1, AC099850.4, PTOV1-AS2, AC245041.2, and AL357033.4.

### The role of the 8-lncRNA signature in HNSCC’s prognosis

The signature score of these 8 lncRNAs based on regression coefficients in multivariable Cox analysis was calculated as follows: signature score = (0.36314 × expression of MIR4435-2HG) + (0.23003 × expression of LINC02541)– (0.22031 × expression of MIR9-3HG) – (0.23426 × expression of AC104083.1) + (0.21344 × expression of AC099850.4) – (0.27806 × expression of PTOV1-AS2) + (0.25463 × expression of AC245041.2) – (0.31513 × expression of AL357033.4). Taking the median signature score as the dividing point, the patients were divided into high signature-score group and low-signature score group. (Figure 2D). Patients in the high-signature score group had a significantly worse OS than those in the low-signature score group (Figure 2E). Besides, the AUCs were assessed for 3years (AUC = 0.740) and 5years (AUC = 0.706) survival (Figure 2F), and the results suggest that the signature can effectively evaluate the prognosis of HNSCC patients.

### Development of a prediction model integrating the 8-lncRNA signature and clinical characteristics

**Figure 3:**
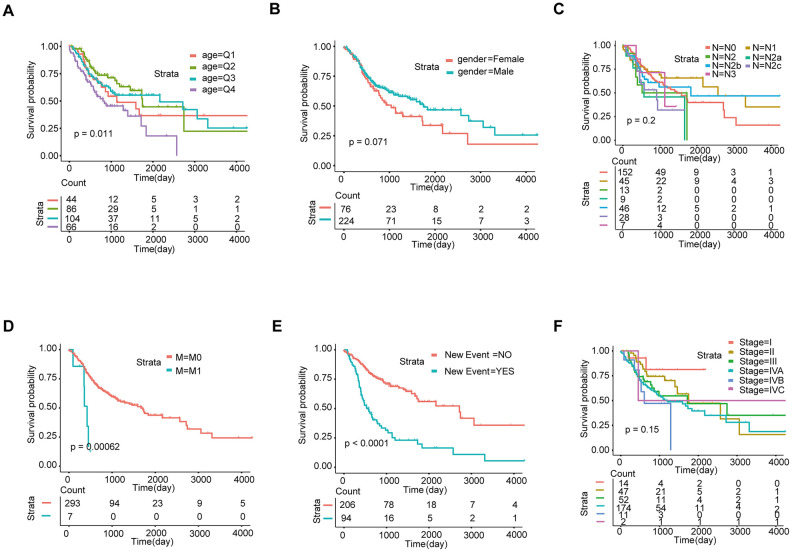
**Screening of prognosis-related clinical characteristics by Kaplan-Meier analysis.** (**A**) Kaplan-Meier curves based on different age groups, where Q1, Q2, Q3, and Q4 represent quartiles. (**B**) Kaplan-Meier curves based on gender. (**C**) Kaplan-Meier curves based on different N stages. (**D**) Kaplan-Meier curves based on different M stages. (**E**) Kaplan-Meier curves based on new events. (**F**) Kaplan-Meier curves based on different tumor stages.

We evaluated age, sex, lymph node (N) status, metastasis (M) status, tumor stage (stage), and new events (which include locoregional disease, locoregional recurrence, new primary tumor, and distant metastasis) using KM analysis. Next, we found that age, metastasis, and new event play an important role in the prognosis of HNSCC (Figure 3).

**Figure 4:**
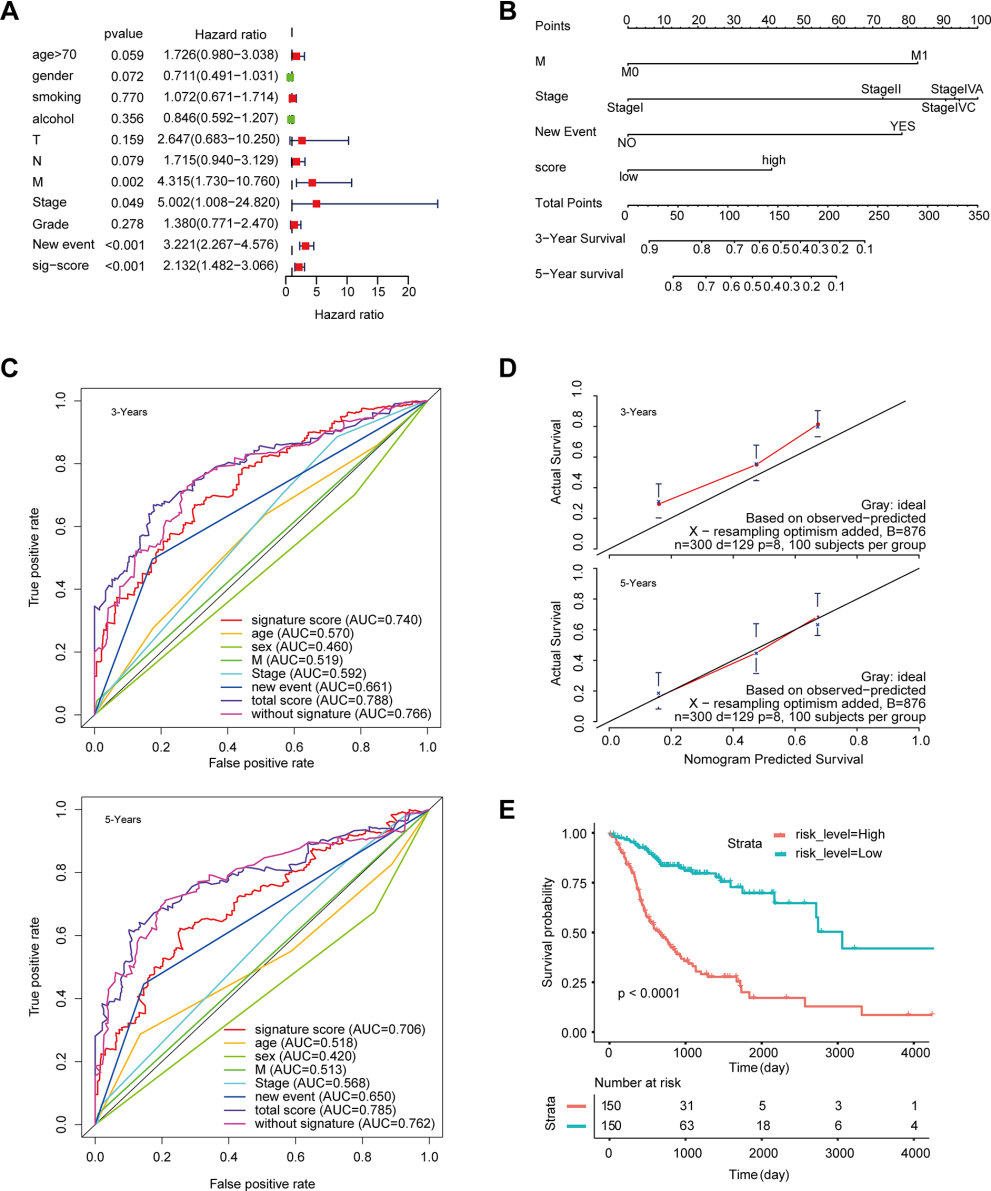
**Construction of a nomogram for overall survival prediction in HNSCC.** (**A**) Univariate and multivariate Cox regression analyses of clinical factors associated with overall survival. (**B**) The nomogram consists of M stage, new event, stage and the signature score based on the eight-lncRNA signature. (**C**) ROC curves according to the nomogram and lncRNA signature score. (**D**) Calibration curves of the nomogram for the estimation of survival rates at 3 and 5 years. (**E**) Kaplan-Meier curves of OS according to the total risk score.

The signature was regarded as a predictor for HNSCC patients. We identified the significant variables through univariate Cox analysis. The multivariate model includes candidate variables with a P-value < 0.1 in univariate analysis. (Figure 4A). Finally, the results (Table 1) suggested that the independent risk factors for HNSCC, including: stage, M stage, new event, and signature score. Moreover, we compared the multivariate Cox regression results of the two groups with and without the signature score. Surprisingly, the C-index of the signature score-containing group (0.72) was higher than that of the signature score-free group (0.71) (Supplementary Figure 1). The nomogram model was built by using the coefficients of the multivariable Cox regression model (Figure 4B). The AUC for 3-year survival reached 0.788 (Figure 4C). What’s more, the calibration curve shows that concerning the probabilities of 3-year OS and 5-year OS, the predicted values are consistent with the observed values (Figure 4D). Finally, we calculated the total risk score based on each predictor in the nomogram model. Kaplan-Meier analysis showed that patients in the high-risk group had a significantly worse OS than those in the low-risk group (Figure 4E).

**Table 1:** The results of multivariate Cox analysis.

	**HR**	**Lower 95%*CI***	**Upper 95%*CI***	***P*-value**
**Age**				
<50y				
50-60y	0.825	0.450	1.511	0.533
60-70y	1.035	0.579	1.849	0.907
≥70	1.652	0.910	3.001	0.099
**sex**				
male vs female	0.696	0.462	1.049	0.083
**N**				
N0				
N1	0.706	0.406	1.228	0.218
N2	1.196	0.498	2.872	0.688
N2a	2.313	0.894	5.989	0.084
N2b	0.754	0.420	1.354	0.344
N2c	1.137	0.593	2.182	0.699
N3	0.388	0.114	1.319	0.129
**M**				
M1 vs M0	3.968	1.287	12.237	0.016^*^
**Stage**				
Stage I				
Stage II	2.535	0.573	11.216	0.220
Stage III	4.328	0.989	18.939	0.052
Stage IVA	4.547	1.078	19.178	0.039^*^
Stage IVB	5.015	0.956	26.299	0.057
Stage IVC	2.949	0.3231	26.922	0.338
**New event**				
yes vs no	3.032	2.081	4.418	<0.001^***^
**signature score**				
high vs low	1.904	1.304	2.780	<0.001^***^

Abbreviations: HR, Hazard ratio; *CI*, Confidence interval; ^*^P<0.05; ^**^ P<0.01; ^***^ P<0.001.

### Validate the signature in the internal and external validation cohorts

**Figure 5:**
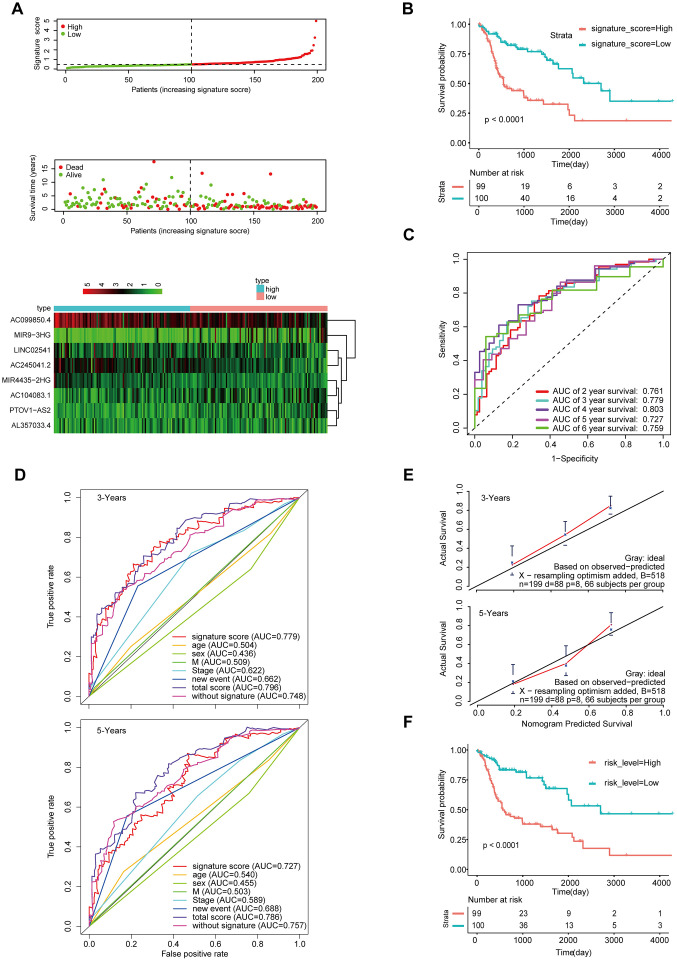
**Validation of the model by the internal validation set TCGA-HNSCC (n=199).** (**A**) Distribution of 8-lncRNA-based signature scores, lncRNA expression levels and patient survival durations in the internal validation set. (**B**) Kaplan-Meier curves of OS based on the 8-lncRNA signature. (**C**) ROC curve analyses based on the 8-lncRNA signature. (**D**) ROC curves according to the nomogram and lncRNA signature score. (**E**) Calibration curves of the nomogram for the estimation of survival rates at 3 and 5 years. (**F**) Kaplan-Meier curves of OS according to the total risk score.

To determine the stability of this nomogram; we performed a similar analysis process in the validation cohort (n = 199). Taking the median signature score as the dividing point, the patients were divided into the high signature-score group (n = 100) and the low signature-score group (n = 99). with the median signature score as the cut-off point (Figure 5A). The Kaplan-Meier OS curves suggested that patients in the high-signature score group had a significantly worse OS than those in the low-signature score group (Figure 5B). The AUC value for 3-year survival exhibited by the 8-lncRNA signature reached 0.779 (Figure 5C). Besides, the calibration curve shows that concerning the probabilities of 3-year OS and 5-year OS, the predicted values are consistent with the observed values (Figure 5E). What’s more, using the same total risk score formula in the internal validation cohort, the Kaplan-Meier OS curves showed that the OS of patients with the high-risk score was significantly worse than that of patients with the low-risk score (Figure 5F). The AUC exhibited by the total risk score for 3-year survival reached 0.796 (Figure 5E).

**Figure 6:**
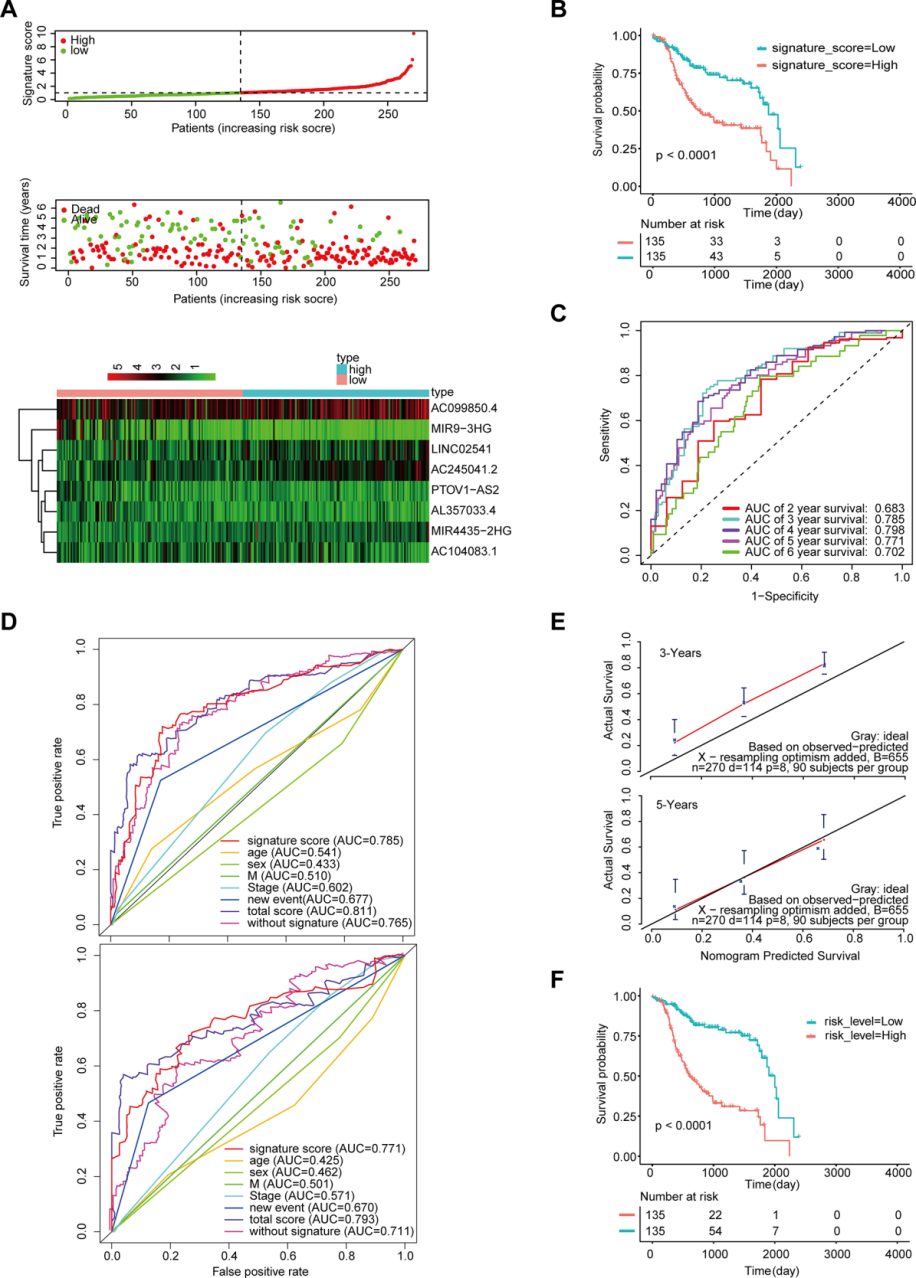
**Validation of the model by the external validation set GSE65858 (n=270).** (**A**) Distribution of 8-lncRNA-based signature scores, lncRNA expression levels and patient survival durations in the external validation set. (**B**) Kaplan-Meier curves of OS based on the 8-lncRNA signature. (**C**) ROC curve analyses based on the 8-lncRNA signature. (**D**) ROC curves according to the nomogram and lncRNA signature score. (**E**) Calibration curves of the nomogram for the estimation of survival rates at 3 and 5 years. (**F**) Kaplan-Meier curves of OS according to the total risk score.

We also validated the robustness of the signature in GSE65858 (n = 270), which had an AUC of 0.785 for 3-year OS (Figure 6A, 6C). Moreover, the OS of patients with high-signature score was worse than those of patients with the low-signature score (Figure 6B). The Kaplan-Meier OS curves manifested that patients in the high total risk score group had a significantly worse OS than patients in the low total risk score group (Figure 6F). Similarly, the calibration curve showed good agreement between the predicted and observed values (Figure 6E), and the AUC exhibited by the total risk score for 3-year survival reached 0.811 (Figure 6D).

**Figure 7:**
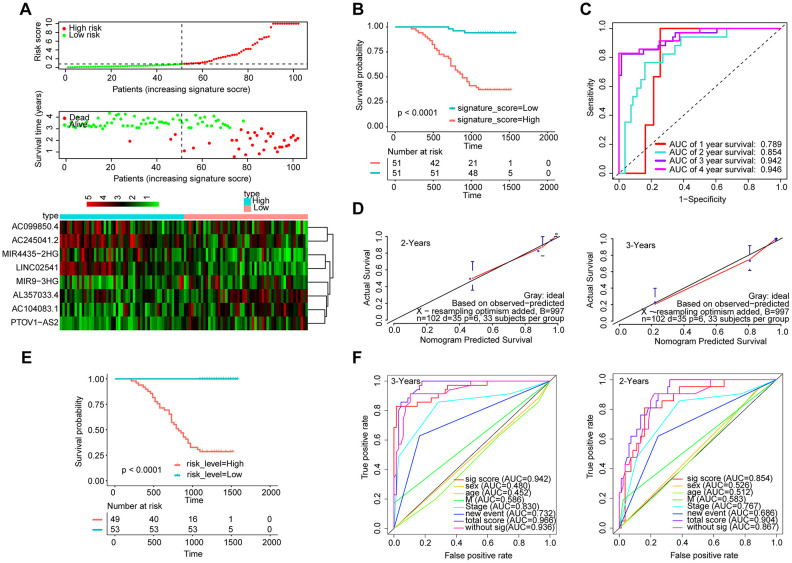
**Validation of the model by the qRT-PCR set (n=102).** (**A**) Distribution of 8-lncRNA-based signature scores, lncRNA expression levels and patient survival durations in the qRT-PCR validation set. (**B**) Kaplan-Meier curves of OS based on the 8-lncRNA signature. (**C**) ROC curve analyses based on the 8-lncRNA signature. (**D**) Calibration curves of the nomogram for the estimation of survival rates at 2 and 3 years. (**E**) Kaplan-Meier curves of OS according to the total risk score. (**F**) ROC curves according to the nomogram and lncRNA signature score.

Furthermore, we measured the expression of these eight lncRNAs in 102 HNSCC samples by qRT-PCR (Figure 7A). The Kaplan-Meier curve showed that the OS of the patients with a high-signature score was significantly worse than that of the patients with a low-signature score (Figure 7B). The AUC for 3-year survival reached 0.942 (Figure 7C). The Kaplan-Meier curve showed that the OS of the patients with a high-risk score was significantly worse than that of the patients with a low-risk score (Figure 7E). The calibration curve performs well (Figure 7D), and the AUC exhibited by the total risk score for 3-year survival reached 0.896 (Figure 7F).

Moreover, the above verification process was also performed for the entire TCGA-HNSC set (n=499) and revealed good results (Supplementary Figure 2).

### WGCNA

**Figure 8:**
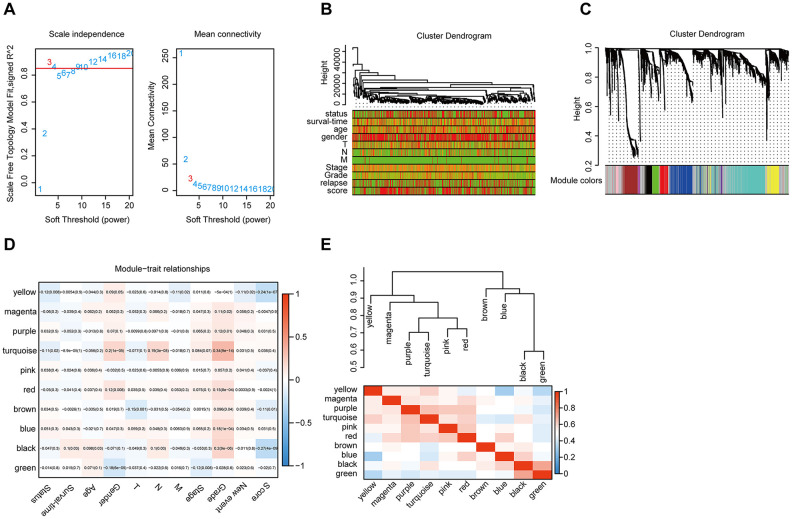
**WGCNA.** (**A**) Analysis of the scale-free topology model fit index for various soft-thresholding powers (β) and the mean connectivity for various soft-thresholding powers. Overall, 3 was the most fitting power value. (**B**) Dendrogram of the genes and different clinical factors of HNSCC (survival time, survival status, sex, age, grade, stage, T stage, N stage, M stage, new event, signature score). (**C**) Dendrogram of the gene modules based on a dissimilarity measure. The branches of the cluster dendrogram correspond to the different gene modules. Each piece of the leaves on the cluster dendrogram corresponds to a gene. (**D**). Module-trait relationships. Heatmap of the correlation between module eigengenes and clinical characteristics of HNSCC. (**E**) Hierarchical clustering and heatmap of the hub gene network.

**Figure 9:**
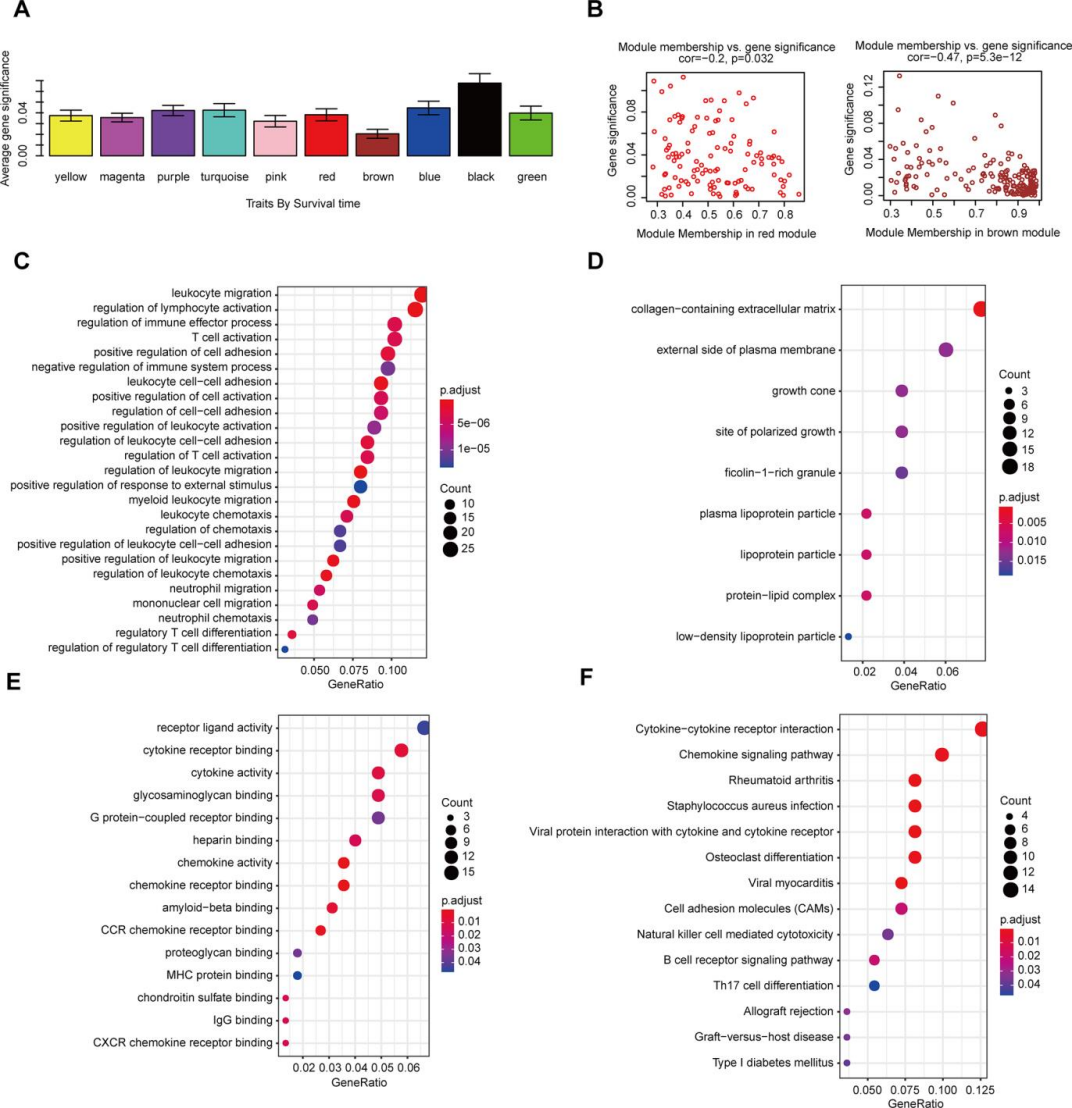
**The correlation between the genes in the modules and survival time.** (**A**) Distribution of mean gene significance and standard deviation with survival time in the HNSCC modules. (**B**) Scatter plot of module eigengenes in red and brown modules. GO (**C**–**E**) and KEGG (**F**) pathway enrichment of eight modules. GO enrichment contains three categories: biological process (**C**), cellular component (**D**) and molecular function (**E**).

The gene co-expression system was established by WGCNA to screen the biologically significant gene modules related to the lncRNAs in the signature. To create a scale-free system, we set the soft threshold beta to 3(Figure 8A). Besides, genes with similar patterns were clustered in different modules (Figure 8B). The minimum cluster size was determined to be 30 per module. The gene modulus was determined by the dynamic shearing method. The module eigengene (ME) was calculated to explore the similarity of all modules (Figure 8C). Eigengenes were calculated to be correlated with clinical factors. Finally, a robust correlation between the gene significance and grade and signature score was identified (Figure 8D). The ten modules were clustered into two groups (Figure 8E). In order to evaluate the correlation between gene expression and survival time, we calculated the gene significance (Figure 9A). Then, we found that there was a strong correlation between the module members of the brown module and the genetic significance of OS. (cor-value = -0.47, *P* = 5.3e − 12). The red module, whose hub gene contains MIR4435-2HG, was also negatively correlated with the OS (cor-value = -0.2, *P* = 0.032) (Figure 9B). Finally, we explore the GO term and KEGG pathway through functional enrichment analysis. (Figure 9C–9F). The results indicated that the biological processes (BP) of these genes mainly involved cell chemotaxis, leukocyte migration, immune response, cell-cell signaling, and so on. The results suggested that the molecular functions (MF) of these genes were related to actin binding, chemokine activity, chemokine receptor binding, ATPase binding, and so on. The results showed that the cellular components (CC) included collagen-containing extracellular matrix, plasma lipoprotein particle, growth cone and site of polarized growth. KEGG pathway functional enrichment showed that leukocyte transendothelial migration, cytokine-cytokine receptor interaction, cell adhesion molecules (CAMs), and the chemokine signaling pathway were mainly related to the genes in these modules.

**Figure 10:**
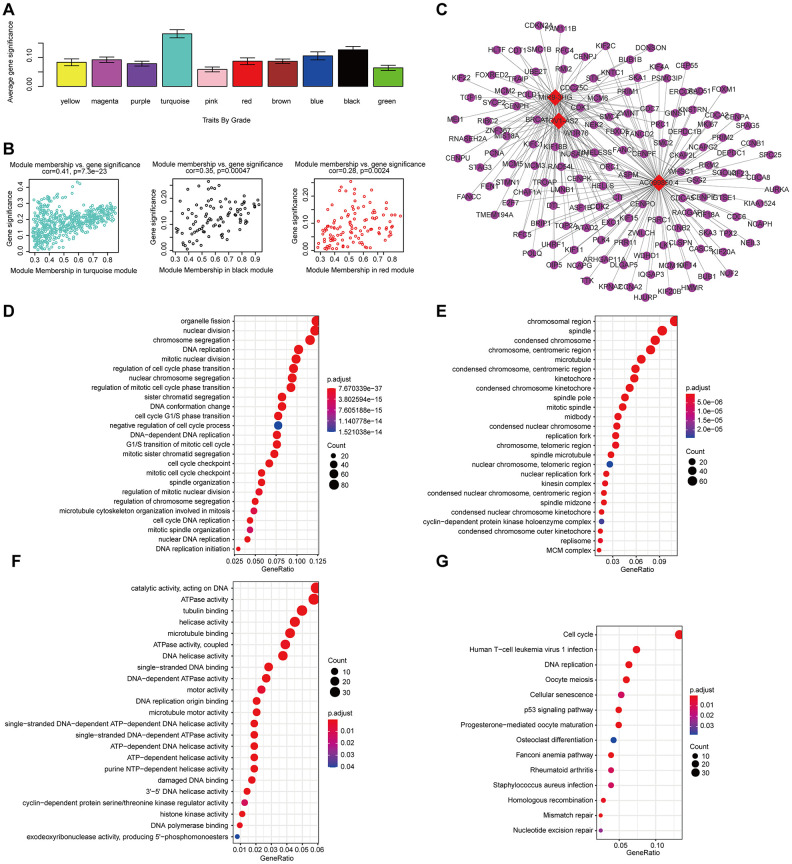
**The correlation between the genes in the modules and grade.** (**A**) Distribution of mean gene significance and standard deviation with grade in the HNSCC modules. (**B**) Scatter plot of the module eigengenes in the turquoise, black, and red modules. (**C**) The lncRNA-mRNA network (weight>0.1) of the hub lncRNAs in the turquoise module. Red and blue diamond shapes represent up- and downregulated lncRNAs, respectively. Purple circles represent mRNAs. GO (**D**–**F**) and KEGG (**G**) pathway enrichment of eight modules. GO enrichment contains three categories: biological process (**D**), cellular component (**E**) and molecular function (**F**).

We conducted a similar analysis process to estimate the correlation between gene expression and grade (Figure 10A). A strong correlation was found between the gene significance for grade and module membership in the turquoise module (which contains MIR9-3HG, AC099850.4 and PTOV1-AS2) (cor-value = 0.41, P = 7.2e − 23); the black module (which contains LINC02541) (cor-value = 0.35, P = 0.00047) and the red module (cor = 0.28, P = 0.0024) were both positively correlated with grade (Figure 10B). We constructed the lncRNA-mRNA network (weight>0.1) diagram of the hub lncRNAs in the turquoise module (Figure 10C). We also carried out functional enrichment analysis to explore the GO term and KEGG pathway (Figure 10D–10G). The results indicated that BP mainly involved cell proliferation, cell division, positive regulation of cell migration, and regulation of the cell cycle. The results showed that MF was related to catalytic activity, acting on DNA, protein binding, and DNA replication origin binding. The results showed that CC included proteinaceous extracellular matrix, chromosome, centromeric region, and extracellular matrix. Moreover, KEGG pathway functional enrichment showed that the cell cycle, the p53 signaling pathway, Cellular senescence, Mismatch repair, and DNA replication were mainly involved.

## DISCUSSION

Head and neck cancer ranks as the sixth leading malignancy worldwide, with almost 90% of cases classified as head and neck squamous cell carcinoma (HNSCC) [6]. Although the diagnosis and treatment have advanced in recent years, HNSCC still has a high incidence and mortality rate in developing countries [3]. Therefore, exploring diagnostic and prognostic biomarkers of HNSCC is urgent.

In the present study, we conducted a difference analysis between tumor and normal tissues in the TCGA-HNSC dataset. Through univariate Cox regression and LASSO analysis, we confirmed that lncRNAs were remarkably correlated with prognosis. Ultimately, eight lncRNAs (MIR4435-2HG, LINC02541, MIR9-3HG, AC104083.1, AC099850.4, PTOV1-AS2, AC245041.2, AL357033.4) were screened to compose a prognostic signature for HNSCC. A robust nomogram consisting of the signature, M, new event, and the stage was constructed for the prognostic prediction of HNSCC patients. Moreover, the AUC value of the signature-based nomogram was better than that of M, new event, and the stage at 3 and 5 years. Besides, In this study, the AUC area analyzed by ROC curve is better than that of similar studies in most HNSCC [7, 8]. The results were verified in the internal validation set, the external validation set, and the qRT-PCR validation set of 102 HNSCC samples.

After a literature review, we found no research had been conducted about the mechanisms of the eight lncRNAs except MIR4435-2HG. MIR4435-2HG is the host gene of MIR4435-2, which is considered to be a biomarker in various cancers, such as oral squamous cell carcinoma [9], non-small-cell lung cancer cells [10], prostate carcinoma [11], gastric cancer [12], hepatocellular carcinoma [13] and lung cancer [14]. MIR4435-2HG promotes cancer cell migration and proliferation mainly by positively regulating TGF-β1 and activating the Wnt/β-catenin signaling pathway [9–14]. Interestingly, we found that the expression level of MIR4435-2HG was positively correlated with the risk score of patients with HNSCC in our study, which was consistent with the results of previously published literature. What is noteworthy is that HNSCC patients with high MIR4435-2HG expression appeared to have a poor prognosis.

To further clarify the mechanism of 8-lncRNAs affecting the survival of HNSCC patients, we selected AC099850.4 and AL357033.4, which showed the most differences in expression, for in vitro experiments. The results show that AL357033.4 overexpression could inhibit the proliferation of HNSCC cell FaDu and Hep-2. Moreover, knockdown of AC099850.4 could suppress the proliferation of FaDu and Hep-2 cells (Supplementary Figure 3). These results suggested that AL357033. 4 and AC099850.4 may be involved in HNSCC proliferation and progression.

Nomograms have been developed in the majority of cancer types. For many cancers, the use of nomograms is more popular than traditional staging systems. [15–17], and thus, it has been proposed as an alternative or even a new standard [18–20]. In this study, a prognostic nomogram combining a lncRNA signature with clinical factors was established. Besides, our nomogram has better prediction accuracy than each factor alone.

We used WGCNA and classified these genes into ten modules according to their expression profiles. Among these modules, we further pay attention to the gene modules that are highly related to various clinical features. Regarding survival time, the functional enrichment analysis indicated that the mRNAs associated with MIR4435-2HG were mainly associated with cellular signal transduction and the chemokine signaling pathway. Interestingly, aside from the feature of grade, the GO terms of the mRNAs that have a close connection with MIR4435-2HG, LINC02541, MIR9-3HG, AC099850.4, and PTOV1-AS2 were mainly focused on cell proliferation, cell division, and cell migration, while KEGG was mostly concentrated on tumor-related pathways such as the p53 signaling pathway, pathways in cancer, the cell cycle and ECM-receptor interaction.

In conclusion, we comprehensively evaluated the risk associated with clinical factors and lncRNAs and their contribution to prognosis and carried out risk stratification. The nomogram proposed in the present study objectively and accurately predicted the prognosis of patients with HNSCC.

## MATERIALS AND METHODS

### Data acquisition

The RNA-sequencing data of HNSCC patients were acquired from The Cancer Genome Atlas (TCGA) database (http://cancergenome.nih.gov/) and The Gene Expression Omnibus (GEO) database (https://www.ncbi.nlm.nih.gov/geo/) [21]. GSE65858 from GEO was conducted on the GPL10558 platform. Besides, we also followed 102 HNSCC patients in the Pathology Department and the Otolaryngology Department of Chengdu Third People's Hospital. The clinical features of patients with HNSCC are presented in Table 2.

**Table 2:** The clinical features of patients with HNSCC.

**Characteristics**	**Training dataset TCGA-HNSC (n=300)**	**Validation dataset TCGA-HNSC (n=199)**	**Validation dataset GSE65858 (n=270)**
**Age (y)**			
< 50	44	31	41
50-60	86	59	112
60-70	104	65	64
> 70	66	44	43
**Gender**			
Male	224	142	223
Female	76	57	47
**Survival status**			
Alive	171	111	94
Dead	129	88	176
**T**			
T1	20	14	35
T2	85	63	80
T3	83	49	58
T4	17	9	-
T4a	91	61	90
T4b	4	3	7
**N**			
N0	152	97	94
N1	45	38	32
N2	13	7	-
N2a	9	7	11
N2b	46	30	66
N2c	28	15	55
N3	7	5	12
**M**			
M0	293	192	263
M1	7	7	7
**Stage**			
I	14	11	18
II	47	33	37
III	52	38	37
IVA	174	114	155
IVB	11	2	16
IVC	2	1	7
**Grade**			
G1	31	32	-
G2	191	114	-
G3	71	52	-
G4	7	1	-
**New Event**			
Yes	94	74	133
No	206	125	137

### Differential analysis

The edgeR package in R software [22] were used to analyze the differentially expressed RNAs in HNSCC and adjacent normal tissues of the TCGA. Significantly expressed RNAs were identified by setting adjusted P values < 0.05 and |log2FC (fold change) | > 1 (|log2FC > 1| and the adjusted FDR < .05) [23, 24].

### The construction of the lncRNA-based prognostic signature

The prognostic value of 253 differentially expressed lncRNAs was first calculated in the univariate Cox analysis, and 41 lncRNAs with P < 0.05 were identified as seed lncRNAs for LASSO regression analysis, which identified 11 lncRNAs (R ‘glmnet’, ‘survival’ packages). To determine the prognostic value of the lncRNAs, multivariate Cox regression was further performed using the R survival package based on each “significant” lncRNA identified in the above steps. A lncRNA with P < 0.05 was defined as significant. The corresponding hazard ratios (HRs), 95% confidence intervals (CIs), and P-values were calculated.

### Prognostic evaluation using the 8-lncRNA signature

The signature score for each patient in the training group is calculated based on the formula (signature score = expGene1 ×βGene1 + expGene2 × βGene2 + expGenen × βGenen (where exp is the prognostic gene expression level and β represents the multivariate Cox regression model regression coefficient)). All samples are randomly divided into high- and low- signature score sets, with the median signature scores as the cut-off value [25]. The survival analysis of each group was evaluated through the Kaplan-Mayer curve and the log-rank test. Receiver operating characteristic (ROC) curve analysis was employed to assess the specificity and sensitivity of the survival predictions according to the lncRNA signature scores (R package “survivalROC”). A *P-*value <.05 was considered significant.

### Development of a prediction model based on the 8-lncRNA signature and clinical characteristics

The gene signature score as a predictor for HNSCC patients was analyzed in the model. We determined the significant variables through univariate Cox regression analysis. The multivariate model includes candidate variables with a P-value < 0.1 on univariate analysis. Finally, the multivariable Cox regression model began with the clinical candidate predictors as follows: stage, M stage, new event, and signature score. The nomogram model was built with the coefficients of the multivariable Cox regression model (using the R packages “rms”, “Hmisc”, “lattice”, “Formula”, and “foreign”). Then, we calculated the total risk score based on each predictor in the nomogram model and divided the HNSCC patients in the training and internal validation sets into two groups with the median risk score as the cut-off point. Kaplan-Meier curves and the log-rank test were used to compare the survival outcomes of the two groups. Receiver operating characteristic (ROC) curve analysis was employed to assess the accuracy and precision. of the survival predictions according to the total risk scores. Calibration curves were plotted to assess the calibration of the nomogram (R package “rms”). To quantify the discrimination performance of the nomogram, Harrell’s C-index was measured. A *P-*value <.05 was considered significant.

### Validation of the 8-lncRNA signature

The same risk formula was used to validate the internal validation set TCGA-HNSC (n = 199), the entire set TCGA-HNSC (n = 499), the external validation set GSE65858 (n = 270) and the qRT-PCR set (n=102).

### Real-time quantitative reverse transcription polymerase chain reaction (qRT-PCR)

Total RNA was reverse-transcribed into cDNA with random primers using the Transcriptor First Strand cDNA Synthesis Kit (Roche, Penzberg, Germany) following the manufacturer’s instructions. The expression levels of the 8 lncRNAs were measured by qRT-PCR using FastStart Essential DNA Green Master mix (Roche, Penzberg, Germany) on a Roche LightCycler 480 (Roche, Penzberg, Germany). Relative expression was determined using inter-experiment normalization to GAPDH. All quantitative PCRs were conducted in triplicate. Divergent primers, rather than the more commonly used convergent primers, were designed for the lncRNAs. Primer specificity was verified using BLAST, with a single peak in the melting curve indicating the generation of a specific product. Three experimental replicates were performed for each sample. Primers used in the study were presented in Supplementary Table 1.

### Construction of a weighted gene coexpression network

The procedure of WGCNA [26] included identifying the gene expression similarity matrix, adjacency matrix, and co-expression network. We set the cut-off as a Person correlation coefficient > 0.9 and P < 0.001 to screen gene coexpression with lncRNAs. Then, differentially expressed gene (DEG) analysis was performed among these genes, and we used the expression matrix composed of 4150 differential genes and the above 8 lncRNAs as input files. The power value of the adjacent matrix soft threshold is determined to be 9 to meet the scale-free topology standard. Hierarchical clustering analysis based on average linkage used the dynamic tree cut method for branch cutting (deep split = 2, cut height = 0.25, minimum cluster size = 30). If the similarity of the modules is > 0.9, they are merged. Based on the level of expression of each gene in each sample, we calculated the correlation between the genes in these modules and the individual phenotypes to measure the correlation between the gene and the phenotype (gene significance). The associations between the modules and variables were assessed to select the relevant modules. The lncRNA-mRNA network visualization was performed via Cystoscope software version 3.7.2 (https://cytoscape.org/) [16].

### Module function annotation

The enrichment analysis was conducted by DAVID [version 6.8] (https://david.ncifcrf.gov) [27] GO consists of three parts: biological processes (BP), molecular function (MF), and cellular composition (CC). Besides, all important GO or KEGG terms or genes are filtered into the meaning of P < .05 and at least two mRNAs associated.

### Ethics statement

As the data (TCGA and GEO datasets) are publicly available, no ethical approval was required.

## Supplementary Material

Supplementary Methods

Supplementary Figures

Supplementary Table 1
