# Supplementary material for: Identification of a nomogram based on an 8-lncRNA signature as a novel diagnostic biomarker for head and neck squamous cell carcinoma
Source: Aging (Albany NY). 2020 Oct 22;12(20):20778–800. doi: 10.18632/aging.104014 (PMC7655182; doi:10.18632/aging.104014)
Supplement: Supplementary Methods [file aging-12-104014-s001..pdf]

SUPPLEMENTARY METHODS

Cell line and culture

FaDu and Hep-2 human laryngeal carcinoma cells were routinely cultured in Dulbecco's modified Eagle's medium (DMEM; Gibco Corporation, Carlsbad, CA, USA) supplemented with 10% fetal bovine serum (FBS; Invitrogen, Carlsbad, CA, USA), 100 U/mL penicillin G, and 100 U/mL streptomycin (Gibco, Carlsbad, CA, USA) in a humidified atmosphere of 95% air and 5% CO2 at 37 °C. The medium was changed every 3 days.

Proliferation assay

FaDu and Hep-2 cells in logarithmic growth phase were seeded in 96-well microplates with 1×10^4 each well. The proliferation of FaDu and Hep-2 cells were assessed by using CCK-8 assay. After 24, 48, 72, and 96 h, cells were treated with 10 μL of CCK-8 reagent (Dojindo Molecular Technologies, Kunamoto, Japan) and incubated at 37 °C for 1 h. An automatic microtiter plate reader was set to zero according to the control wells. The absorbance (*A*) of each well was measured at a wavelength of 450 nm.

lncRNA knockdown and overexpression

Small interfering RNAs (siRNAs) of AC099850.4 and overexpression of AL357033.4 plasmid were constructed by GenePharma (Shanghai, China). Cells were transfected using Lipofectamine 2000 (Invitrogen, CA, USA). After 48 h of siRNA knockdown or plasmid transfection, lncRNA expression was measured using qRT-PCR.
