# Supplementary material for: Identification of a nomogram based on an 8-lncRNA signature as a novel diagnostic biomarker for head and neck squamous cell carcinoma
Source: Aging (Albany NY). 2020 Oct 22;12(20):20778–800. doi: 10.18632/aging.104014 (PMC7655182; doi:10.18632/aging.104014)
Supplement: Supplementary Figures [file aging-12-104014-s002..pdf]

SUPPLEMENTARY FIGURES

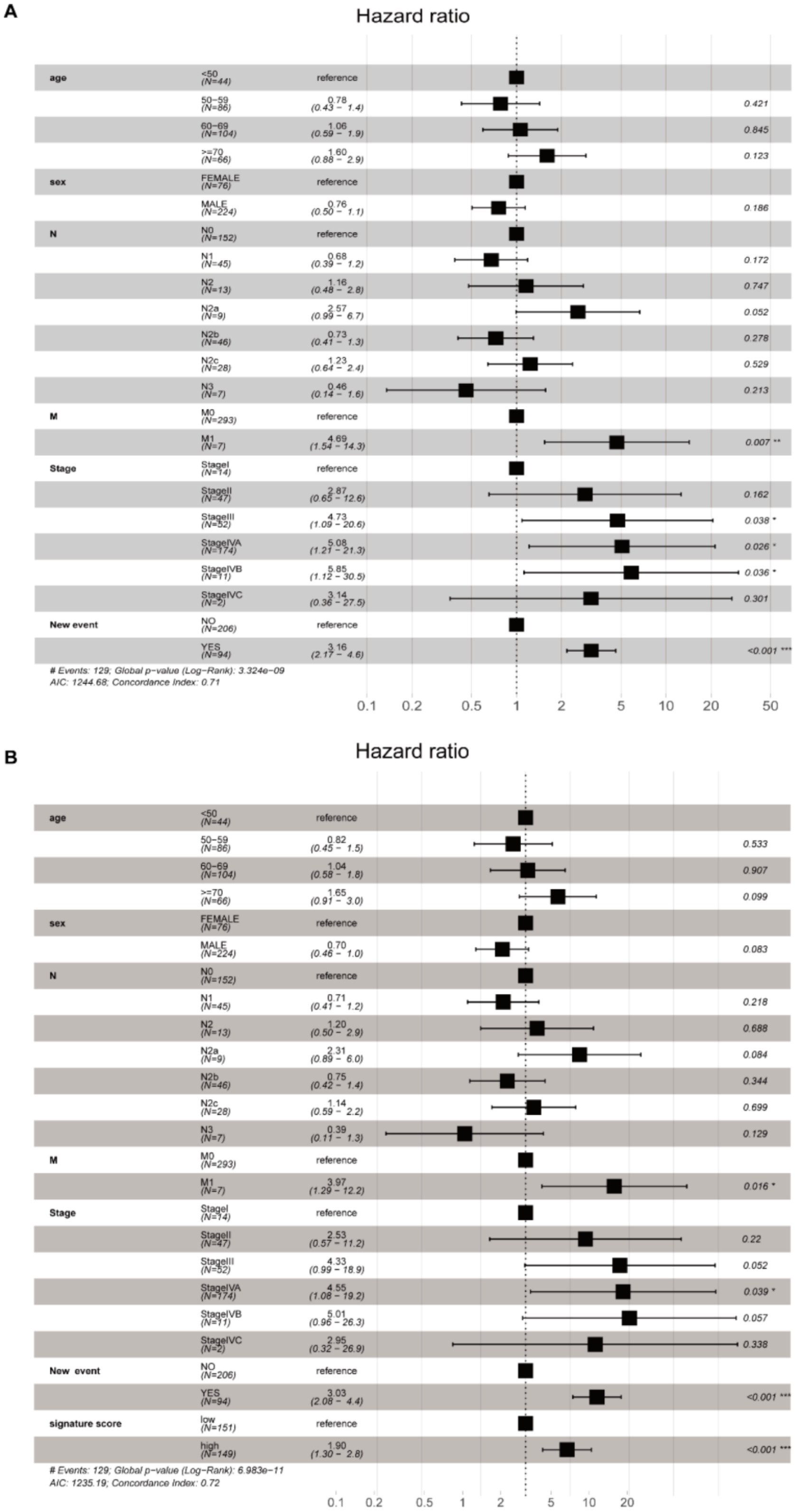

Supplementary Figure 1. Comparison of the multivariate Cox regression results of the two groups with and without the signature score. (**A**) The group without the signature score. (**B**) The group with the signature score.

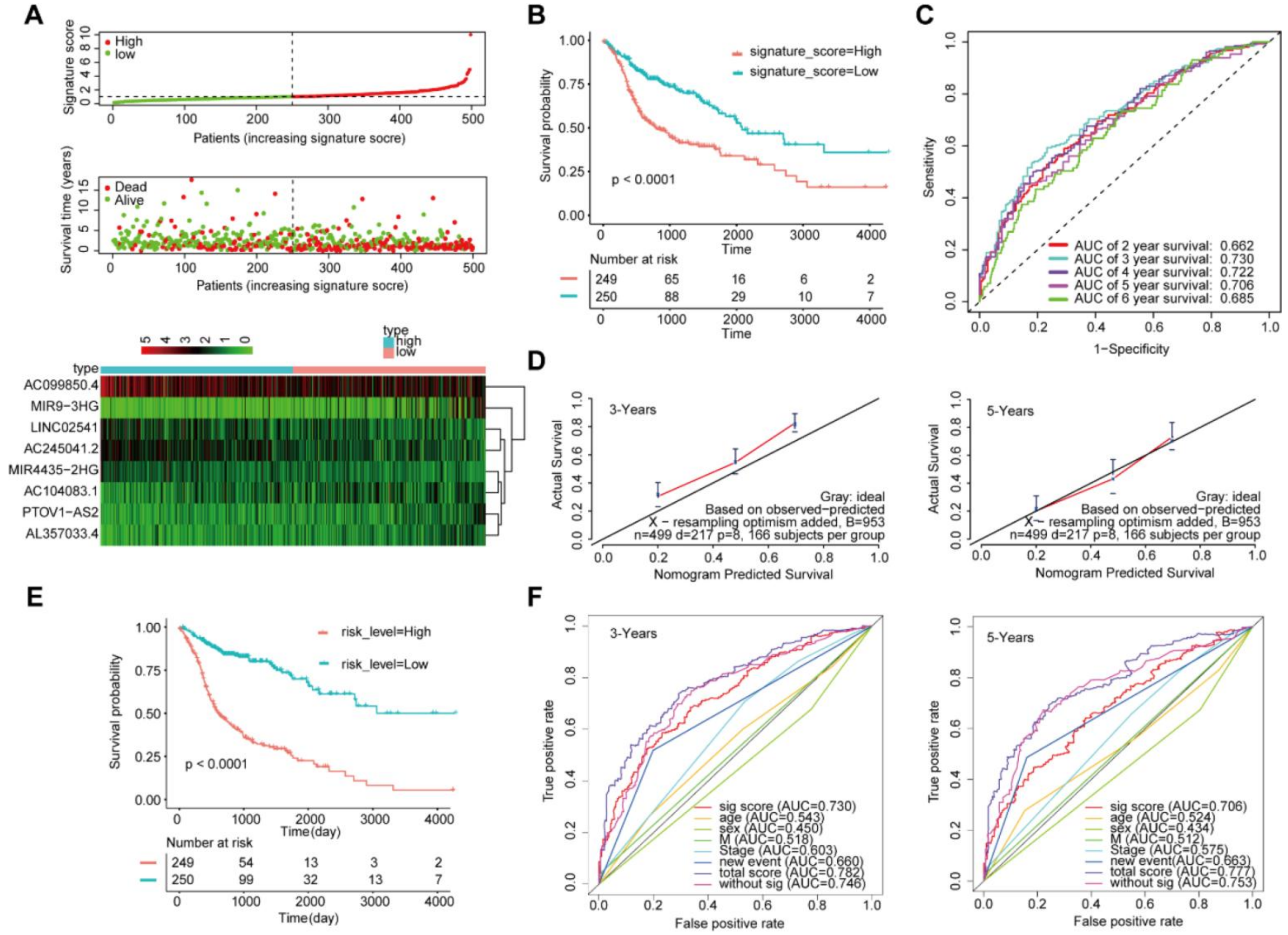

Supplementary Figure 2. Validation of the model by the entire TCGA-HNSCC set (n=499). (**A**) Distribution of 8-lncRNA-based signature scores, lncRNA expression levels and patient survival durations in the entire set. (**B**) Kaplan-Meier curves of OS based on the 8-lncRNA signature. (**C**) ROC curve analyses based on the 8-lncRNA signature. (**D**) Calibration curves of the nomogram for the estimation of survival rates at 3 and 5 years. (**E**) Kaplan-Meier curves of OS according to the total risk score. (**F**) ROC curves according to the nomogram and lncRNA signature score.

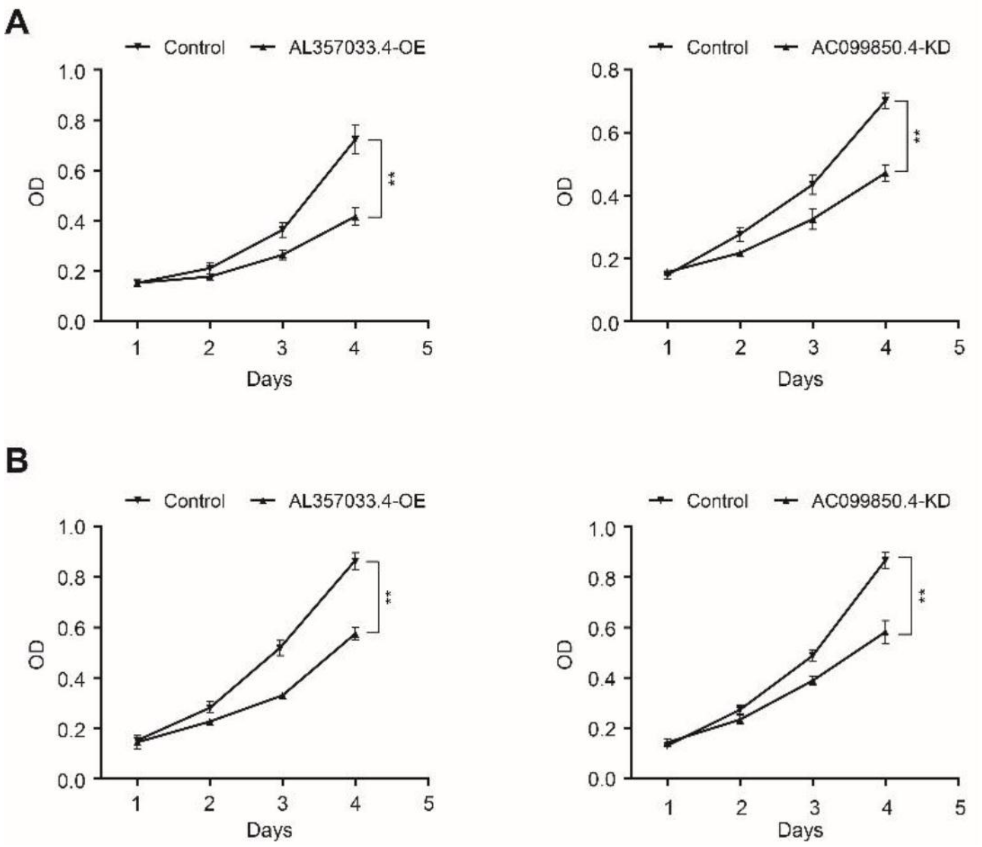

Supplementary Figure 3. Proliferation of FaDu (**A**) and Hep-2 (**B**) cells was analyzed using CCK-8 assay following lncRNAs overexpression or knockdown.
