# Supplementary material for: Identification of a nomogram based on an 8-lncRNA signature as a novel diagnostic biomarker for head and neck squamous cell carcinoma
Source: Aging (Albany NY). 2020 Oct 22;12(20):20778–800. doi: 10.18632/aging.104014 (PMC7655182; doi:10.18632/aging.104014)
Supplement: Supplementary Table 1 [file aging-12-104014-s003..pdf]

SUPPLEMENTARY TABLE

Supplementary Table 1. Primers used in the study.

Gene	Sequence	Product length (bp)
MIR9-3HG	5’-GCCAGGCTTACTGTCTCTGG-3’	170
	3’-GAGCCAAGACCTATCCACCA-5’	
AC099850.4	5’-TCACCATGCCTGGGTAATTT-3’	151
	3’-TGCCAAGGAATCTCTGAAGTC-5’	
MIR4435-2HG	5’-CATTTTTCCCTGCTCTGCTC-3’	151
	3’-ATCAAGGAGGGCTCATGTTG-5’	
AC104083.1	5’-AGCCTTCCAGATTGTGAGGA-3’	209
	3’-ACATTGGGCTGTCCAACTTC-5’	
AC245041.2	5’-AGGAGCCAGACATGTGGAGT-3’	208
	3’-CAGAGCTTCTGCTGTGCAGT-5’	
AL357033.4	5’-TCGGTCATCAGTTCCATCAA-3’	166
	3’-TAGCAGTGAACGCAGAGGTG-5’	
LINC02541	5’-CCAGGCTGGAGTATGATGGT-3’	160
	3’-TGGTGAAACCCCGTCTCTAC-5’	
PTOV1-AS2	5’-CTGTCCCTGAGGAGTGGAGA-3’	178
	3’-AGCGGTGGAGATGACGTTTC-5’	
GAPDH	5’-GAAAGCCTGCCGGTGACTAA-3’	150
	3’-GCCCAATACGACCAAATCAGAG-5’	
